# The International Medical Annual and Practitioner’s Index

**Published:** 1896-05

**Authors:** 


The International Medical Annual and Practitioner's Index. A work of reference for Medical Practitioners. Fourteenth year. Price, $2.75. New York: E. B. Treat, 5 Cooper Union. 1896.

The 1896 volume of the annual comes at its regular time and like its predecessors is eagerly welcomed by the profession. The general make-up of the 1896 number is similar to the preceding volumes and contains 728 pages of solid matter, every page of which is good reading. The therapeutic review of the past year, by Professor H. A. Hare, comprises short sketches on anesthetics, strychnia, chlorate of potassium and acetanilide.

The dictionary of new remedies by various European and American authors comprises fifty-eight pages, general anesthetics, chloralose and uranium receiving quite extended notice. The special essays include a very concise report on the parasite of malaria, its biology and method of detection, including a colored plate. The article on diagnosis of toothache and neuralgia of dental origin is timely and is ably written by Henry Sewill, M. R. C. S. A very interesting article to many physicians is that on the remedial value of cycling, by Oscar Jennings, M. D., M. R. C. S., of Paris. The value of cycling to women receives important consideration.

The article on sensory distribution of spinal nerve roots, by William Thorburn, is an important addition to our knowledge of nerve distribution and sensory areas. The article is accompanied by a double-page colored plate.

The article on angio-neurosis is well presented by W. Ramsay Smith and is also accompanied by four excellent illustrations. The article on life assurance is from the pen of F. DeHaviland Hall, physician to the Westminster hospital. He considers carefully the personal history, family history, present condition and environment of the assured individual.

The dictionary of new treatment in medicine and surgery embraces many excellent monographs, especially on diphtheria treatment, diseases of the ear, eye, bladder, heart, kidney, larynx, treatment of obstinate hiccough, intestinal surgery, leucorrhea and the like. The new photography receives careful attention, including several plates of photographs by E. Henry Fenwick.

The concluding chapters treat of inventions in sanitary science, new medical and surgical instruments, new books published during the year and a very complete index.

To those who have the former volumes this one needs no words of recommendation, but to those physicians who do not possess the former volumes we advise to begin with the 1896 volume. The book is bound similar to the volumes in the series of Treat's classics. W. C. K.

